# Sleep-Related Factors in Shift Workers: A Cross-Sectional Cohort Pilot Study to Inform Online Group Therapy for Insomnia

**DOI:** 10.3390/ijerph22111681

**Published:** 2025-11-06

**Authors:** Tanja Grünberger, Christopher Höhn, Manuel Schabus, Anton-Rupert Laireiter

**Affiliations:** Department of Psychology, Paris Lodron University Salzburg, 5020 Salzburg, Austria; christopher.hoehn@plus.ac.at (C.H.); anton.laireiter@plus.ac.at (A.-R.L.)

**Keywords:** shift work, insomnia, tailored treatment, personality traits, psychological stress, sleep-related factors

## Abstract

Shift workers face a heightened risk of insomnia. Recent research has yielded promising insights, but further progress is necessary to better treat insomnia in this group. The present pilot study evaluates how different characteristics impact sleep in shift workers to develop an innovative therapeutic approach. An online survey was administered to an ad hoc sample of *N* = 225 (112 shift workers), and correlations were calculated between sleep variables and specific characteristics (e.g., psychological impairment, personality traits, sleep-related behavior, attitudes towards sleep and shift work). Group differences between good/poor sleepers and day/shift work were determined using Mann–Whitney U-tests and Kruskal–Wallis H-tests. Regression was used to identify appropriate predictors. All factors (except perfectionism, chronotype, and importance of sleep) yielded significant results in both correlations and group differences (good/poor sleepers). The two groups of day/shift workers showed only minor differences. Dysfunctional beliefs about sleep, pre-sleep arousal, and depression were identified as predictors of poorer sleep. We conclude that interventions on psychological constraints (anxiety and depression), personality traits (anxiety, concern, emotional instability, and tension), social integration, sleep-related factors (dysfunctional beliefs, especially cognitive pre-sleep-arousal, sleep hygiene) and the attitude toward shiftwork, can replace those on regularity and will build an innovative therapy for shift workers on this basis. Once the newly developed treatment manual is finalized, its efficacy will be assessed through a randomized controlled trial.

## 1. Introduction

Although shift work affects approximately 20% of the workforce in developed countries [1], and its well established association with a higher prevalence of insomnia, this demographic has long been underrepresented in insomnia research [2,3].

According to the S2k guideline of the German Society for Occupational and Environmental Medicine [4], shift work involves employment at varying times of day or night, or at consistent but atypical hours that differ from regular daytime work. In Germany, “night work” is legally defined by Section 2 of the Working Hours Act as work that includes more than two hours between 11:00 p.m. and 6:00 a.m. However, definitions of night work differ significantly across countries and in scientific literature [4].

A basic distinction is made between permanent (e.g., continuous night shift) and alternating shift systems. In the latter, employees work rotating or irregular shifts, e.g., early and late shifts (two-shift system) or early, late, and night shifts (three-shift system) [4]. The hours covered per shift also vary considerably depending on the branch or organization. Importantly, these descriptions do not include 12 or 24 h shifts, which are common in the medical sector and split shifts (e.g., 6–10 a.m., long break, 4–8 p.m.), which are typical in care facilities or in the tourism sector.

Shift work has been linked to numerous health risks, including cardiovascular diseases, cancer, and increased mortality [5,6,7]. Psychological effects include higher rates of depression, anxiety, suicidal ideation, irritability, nervousness, and burnout [5,8,9,10,11,12]. Reduced job satisfaction, social isolation, work–life imbalance and impaired cognitive performance are also common [8,11,13]. Shift work has further been shown to negatively affect total sleep time (TST, [14]) as those working in three-shift systems show an elevated risk of less than six hours of sleep compared to day workers [15]. Additional effects have been shown regarding prolonged sleep onset latency (SOL), increased rapid eye movement sleep (REM) and non-REM phase 2 (NREM2) sleep, and more frequent and premature sleep interruptions. These sleep disruptions can also be affected by social activities, such as rising to share meals with family members [10] and more challenging sleep conditions during the day [16].

Insomnia is defined as dissatisfaction with sleep quality or quantity, alongside daytime impairment, with a prevalence of 10% [1,17]. Shift work sleep disorder (SWD) is defined as insomnia and/or excessive sleepiness due to the engagement in shift work [1,18], but can also persist on days off or on vacation [19]. SWD prevalence is estimated to be 26.5% among shift workers [18], varying strongly across shift systems (38.8% with three shifts, 24.7% with two, 5.5% with day only) [20]. Although primary insomnia and SWD have different etiologies, a strict diagnostic separation is almost impossible in practice. They often overlap symptomatically and in terms of maintaining factors [21], since SWD frequently evolves into chronic insomnia [9,19,21].

Sleep disturbances in shift workers are typically attributed to a work-related misalignment of the sleep–wake rhythm with the internal circadian system [9,22,23]. This misalignment precipitates the release of melatonin and cortisol at times when their effect is incongruent with the individual’s current objective (e.g., working or sleeping) [5].

Other factors also contribute to the development and persistence of insomnia, and this may be equally true in shift workers. For instance, in a sample of night shift workers, disparities were identified between individuals with adequate sleep and those affected by SWD. These disparities were evident in more negatively toned pre-sleep cognitive activities, dysfunctional beliefs about sleep related to worry/helplessness, and selective attention to sleep [5]. Furthermore, a clear correlation exists between the risk of SWD and the severity of depression [24]. However, some researchers have also questioned the causal role of shift work in health outcomes, citing methodological issues and confounding lifestyle factors [25]. Thus, a broader, multifactorial understanding of sleep disturbances in shift workers is warranted.

CBT-I is the gold standard for treating insomnia [26], but its core components—such as sleep restriction and stimulus control—are based on regularity, which does not apply to irregular schedules that are at the core of shift work. Unfortunately, a common solution for this problem is offering sleeping pills, which exacerbates the issue and might result in resignation or skepticism regarding therapeutic interventions, therefore contributing to the observed decline in compliance [27].

Recent studies suggest that slightly adapted forms of CBT-I are more effective, but that these adaptations alone are not sufficient [27,28,29]. Therefore, there is a clear need for a novel, shift work-specific therapeutic approach.

Consequently, it may be reasonable to replace interventions that require strict regularity with alternative approaches. Based on our literature review, the following factors should be examined: Psychological impairment (anxiety and depression), personality traits (anxiety, concern, emotional instability, tension, and perfectionism), social integration, occupational factors (effort, reward, overcommitment, and imbalance), cognitive factors (dysfunctional beliefs, pre-sleep-arousal, attitude toward shiftwork, and importance of sleep), and also sleep hygiene and chronotype.

While the factors presented here are known from the literature to be related to sleep and sleep disorders, only a few of them have been empirically tested on shift workers. Therefore, this pilot study aims to evaluate the relevance of these factors for shift workers’ sleep, i.e., sleep quality, insomnia severity, total sleep time, sleep onset latency, and daytime sleepiness.

From this general objective, three specific, measurable objectives were derived, and corresponding research questions and hypotheses were formulated.

(RQ1) Which factors of interest are significantly associated with sleep in a sample of shift workers?

**H1.**  *Significant correlations exist between poorer sleep and higher levels of all factors of interest except lower perceived social integration and a more negative attitude towards shift work.*

(RQ2) Do good/poor sleepers and day/shift workers differ significantly regarding the factors of interest?

**H2.**  *Poor sleeping shift workers show significantly higher levels of all factors of interest, except lower perceived social integration and a more negative attitude towards shift work in comparison to good sleeping shift workers and day workers in general.*

(RQ3) Which of the identified significant factors are suitable predictors for sleep in shift workers?

**H3.**  *The highest explained variance is found in psychological impairment, perfectionism, social integration, dysfunctional beliefs, and the importance of sleep.*

## 2. Materials and Methods

### 2.1. Study Design, Recruitment, Participants, and Group Allocation

An online survey was conducted with an ad hoc sample from the general population. To obtain four groups of approximately equal size (good/poor sleep; day/shift work), participants were requested to indicate their perceived group affiliation at the commencement of the survey. When the desired group had already reached maximum capacity, a message was displayed indicating that further participation was not possible.

Participants were recruited from April to October 2021 in the German-speaking region. Companies and institutions with shift work were contacted and invited to forward the survey to their employees. Furthermore, a multifaceted recruitment strategy was employed, encompassing the utilization of mailing lists and social media platforms.

The inclusion criteria encompassed individuals between the ages of 18 and 65 years, with a minimum of 30 working hours per week, and adequate German language proficiency.

Individuals with a history of diagnosed physical illnesses affecting sleep (e.g., restless legs syndrome, sleep apnea syndrome, chronic pain) or other primary disorders (acute substance dependence, severe affective or anxiety disorders, psychoses) were excluded from the study.

No further inclusion or exclusion criteria were applied, as we aimed to study four groups: good vs. poor sleepers, as well as shift vs. day workers. Both day workers and good sleepers were included in order to identify group differences or potential vulnerability/resilience factors.

The Ethics Committee of the University of Salzburg has decided that an exception is granted for this study and no vote by the Ethics Committee is necessary.

An a priori power analysis was conducted using G*Power 3.1 (Düsseldorf, Germany) [30]. Assuming a medium effect size, a power of (1 − β) = 0.95 can be achieved with a total sample size of *N* = 200 in all planned evaluation procedures.

The final sample size comprised 225 participants (59.11% female) with a mean age of 34.88 years (*SD* = 12.92). The criterion for assignment to the shift work group was the regular performance of at least two different shifts (e.g., early and late shift); but most participants also indicated night or split shifts.

The allocation of subjects to the good and poor sleep groups was based on a median dichotomization *Md* = 9 of the ISI (insomnia severity index, [31]). We used this approach as clinical diagnostics could not be performed in an online format, and using the ISI threshold for clinical insomnia of >15 would have resulted in strongly unequal group sizes.

### 2.2. Method and Instrument

The study was conducted online via LimeSurvey, and a standardized instrument was compiled from various validated questionnaires and a few in-house developed items. The survey commenced with questions regarding demographics and previous illnesses, and a question regarding attitudes toward shift work was included (“Do you like working shifts?”—”yes, I don’t mind”; “no, but I have to”).

The total score of the PSQI (Pittsburgh Sleep Quality Index [32]) was employed to assess overall sleep quality, while single items were used to assess sleep onset latency (item 2), and total sleep time (item 4).

The ISI (Insomnia Severity Index [31]) was utilized as an indicator of the severity of insomnia, while the ESS (Epworth Sleepiness Scale [33]) was employed to assess daytime sleepiness. The German version of the DBAS-16 (dysfunctional beliefs about sleep; Ger.: MZS, Meinungen-zum-Schlaf-Fragebogen [34]) was used to gather data on sleep-related dysfunctional beliefs, and the rCSM (reduced Composite Scale of Morningness [35]) was used to assess chronotype. The PSAS (Pre-Sleep Arousal Scale [36]) was utilized to evaluate cognitive and physiological arousal before sleep. To assess sleep hygiene behavior, the SHI (Sleep Hygiene Index [37]) was employed. Finally, a self-developed item regarding sleep importance was presented (‘How important is your sleep to you?’) to gather evidence for the attention-intention-effort-pathway [38].

To examine the factors derived in the introduction for their relationships with sleep and their relevance for shift workers, existing questionnaires were used in whole or in part. The HADS-D (Hospital Anxiety and Depression Scale [39]) was employed to assess psychological well-being, in particular anxiety and depression. The 16 PF-R (16 Personality Factor Test, revised version [40]) was also employed, with subscales measuring emotional instability, tension, concern, and perfectionism as well as the global scale anxiety. The Social Integration subscale from the F-SozU (Questionnaire on Social Support [41]) was also integrated, as well as the ERI (effort-reward-imbalance [42]) to measure work-related personality traits (effort, reward, overcommitment, also contains questions on psychological detachment). All tests exhibited acceptable to good test quality, as assessed according to [43] and displayed in [44].

The survey instrument took approximately 30 min on average to complete.

### 2.3. Statistical Analyses

Statistical significance was reached with an error probability of *p* < 0.05, two-sided. Pearson, Spearman, or biserial correlations were calculated with the sub-sample of shift workers (*n* = 112), depending on data distributions, to test which factors showed a significant correlation with the sleep variables insomnia severity, sleep quality, sleep onset latency, total sleep time, and daytime sleepiness (RQ1).

Group differences (RQ2) were assessed using Kruskal–Wallis H-tests and Mann–Whitney U-tests. η^2^ was calculated as the effect size. Finally, all resultant relevant factors were subjected to multiple linear regression (forced entry, forward). For the sleep variables: insomnia severity, sleep quality, sleep onset latency, total sleep time, and daytime sleepiness, individual regressions were calculated for shift and day workers (RQ3).

After determining that the prerequisites for regression were fulfilled, a preselection had to be made from the eligible independent variables to avoid over- or under-adjustment of the resulting model [45]. On the one hand, this concerned the number of predictors in relation to the sample size [46] and, on the other hand, content-related logical considerations. In order to circumvent redundancies, the total scores of the HADS-D and the PSAS were not integrated, but rather their subscales. The ERI effort and reward subscales are included in the ERI Imbalance measure, so this scale was used. The overcommitment subscale was included separately. Further exclusions of variables were then made on the basis of the results for RQ1 and RQ2.

The decision to initiate the whole analysis of the study with bivariate procedures (correlations, group differences), followed by multiple analyses (regression), may initially appear to be redundant. However, this approach was adopted due to the exploratory nature of the study. While recent research has increasingly focused on shift workers, the number of studies in this area remains limited. Consequently, it is possible that the observed relationships differ from those assumed based on known effects in the general population. The restriction of the analysis to regressions might have precluded the identification of unexpected outcomes.

For RQ1 and 2, the α-level of the results was adjusted [47,48]. The interpretation of the correlation coefficients and effect sizes follows the recommendations of Cohen [49]. All calculations were conducted using SPSS 30 [50].

## 3. Results

The total sample consisted of 225 participants. The distribution values by group are displayed in Table 1.

### 3.1. RQ1: Do the Factors Under Investigation Correlate with Sleep in a Sample of Shift Workers?

The majority of the correlations between the sleep of shift workers and the analyzed characteristics demonstrated significant relationships, particularly for the severity of insomnia (ISI [31]) and sleep quality (PSQI total, [32], see Table 2). The effect sizes ranged from moderate to high, with the most pronounced effects observed in total psychological impairment, emotional stability, and total pre-sleep arousal.

No significant correlations were found between the sleep of shift workers and either perfectionism or the perceived importance of sleep. Later chronotypes exhibited a longer sleep onset latency, all other correlations between chronotype and sleep variables were not significant.

### 3.2. RQ2: Do Good/Poor Sleepers and Day/Shift Workers Differ with Regard to the Factors Under Investigation?

Irrespective of shift or day work, good and poor sleepers differed significantly on all factors tested, again with the exception of perfectionism, chronotype, and importance of sleep. The effect sizes for the difference in insomnia severity were large, as these scores were used for median dichotomization (see Section 2.1). Large effects were also found for total sleep time, psychological impairment in general, and all facets of pre-sleep-arousal. Medium effect sizes were found for sleep onset latency, the states of anxiety and depression, emotional instability, and dysfunctional beliefs. All other examined variables show moderate or medium effects (Table 3).

In contrast, for all variables analyzed, the groups shift/day work only differed from each other with a small to medium effect size for the total sleep time, *Z* = −4.08, *p* = 0.026, η^2^ = 0.07.

The multiple group comparisons (Table 4) also show predominantly significant group differences, with the exception of perfectionism, chronotype, sleep hygiene, and importance of sleep. Large effect sizes were found for total sleep time, insomnia severity, sleep quality, dysfunctional beliefs about sleep and pre-sleep arousal. Sleep onset latency, psychological well-being, and the traits anxiety and emotional instability showed medium effects, all others small effects.

Subsequent post hoc tests revealed that the manifestation of the characteristics differed between the poor (groups 1, 3) and the good sleepers (groups 2, 4); the day (groups 1, 2) vs. shift workers (groups 3, 4) did not.

The distribution of the means shows a clear pattern. The factors examined as possibly associated with lower well-being and greater psychological stress are more pronounced in groups 1 and 3, while groups 2 and 4 are less affected, as shown by the color coding.

### 3.3. RQ3: Which of the Significant Factors Are Suitable Predictors for Sleep in Shift Workers?

Integrating the results of RQ1 and RQ2, perfectionism, chronotype, and importance of sleep showed negligible or nonsignificant group differences or correlations with the sleep variables. Therefore, these variables were not included in the regression analyses, as were variables that would lead to redundancies, as explained in the methods section. The independent variables that were included are the sleep-related dysfunctional beliefs, cognitive and somatic pre-sleep arousal, and sleep hygiene; the states depression and anxiety; the traits emotional instability, tension, concern; the work-related factors imbalance, overcommitment, attitudes toward shift work; and social integration. The requirements for multiple linear regression were met; no outliers needed to be excluded.

The regression models calculated for five sleep variables, with separate analyses for day and shift workers, yielded significant results (Table 5). Insomnia severity and sleep quality exhibited a high variance explanation in both groups. For sleep onset latency, this was only true for the shift workers. The models for all other sleep variables explained moderate to medium variance. For insomnia severity and sleep quality, three independent variables each were integrated, while sleep onset latency consists of two and only one independent variable was used in the models for total sleep time and daytime sleepiness.

The comparison between day and shift workers revealed several similarities, as well as several notable differences. Imbalance was incorporated into the model exclusively for day workers, manifesting in insomnia severity, sleep quality, and total sleep time. Instead, for shift workers, depression manifests in the regression model for insomnia severity, and emotional instability in sleep quality and total sleep time. Depression is relevant in the sleep onset latency model for shift workers, but not for day workers. Dysfunctional beliefs are the only model components for daytime sleepiness in both groups.

## 4. Discussion

The primary conclusions of this study demonstrate that our hypotheses have been predominantly substantiated. In the group of shift workers, the observed correlations between the factors examined and the sleep variables corresponded to the expected direction. That is, poor sleep was associated with manifestations of the factors examined that were indicative of higher stress or lower well-being.

The exceptions to this were perfectionism, chronotype, and the importance of sleep, which did not reach statistical significance. The aforementioned exceptions are equally applicable to the second hypothesis.

The second hypothesis was not entirely supported by the data, but significant group differences were identified for most factors between individuals with good and poor sleep. As anticipated, the mean values of the poor sleep group exhibited a more pronounced inclination toward elevated stress levels. Contrary to prevailing assumptions, however, only negligible differences were identified between day and shift workers.

Therefore, the findings of the regression analyses were equivocal. Depending on the group and the respective dependent variable, different independent variables were integrated into the respective models. In summary, dysfunctional beliefs about sleep, cognitive arousal before falling asleep, and depression contribute to explaining the variance. Emotional instability and imbalance should be considered suitable predictors, albeit with limitations.

The cognitive insomnia model posits that excessive negatively toned cognitive activity triggers arousal and distress, heightens selective attention and monitoring, and distorts the perception of sleep. As a result, individuals increasingly overestimate the importance of sleep, which further elevates anxiety [52]. The attention-intention-effort pathway posits that an excessive focus on sleep and its initiation can then induce and maintain sleep disorders [38].

Both models should of course also apply to shift workers. Recent studies [5,29] explicitly recommend the integration of cognitive interventions into the therapy of shift workers.

However, our results are ambivalent. Cognitive pre-sleep arousal is significant regarding correlations and group differences, but the importance of sleep is not. Since the latter is a self-reported single item, its interpretive weight is limited. Cognitive interventions such as psychoeducation on the development and maintenance of sleep disorders should be integrated into the new therapy accordingly.

Improving sleep hygiene is considered an effective intervention in CBT-I [2]. However, some authors argue that poor sleep hygiene is associated with insomnia, although it is rarely identified as its cause [53,54,55,56]. Due to its emphasis on regularity, sleep hygiene is also challenging for shift workers. We considered that adherence to these rules throughout the day may potentially lead to a heightened focus on sleep, which, according to the attention-intention-effort pathway [38], could result in maintenance of the sleep problem and be therefore maladaptive.

We further hypothesized that sleep hygiene, with its emphasis on regularity, would not be feasible for shift workers. Consequently, we expected a significant difference between shift and non-shift workers, but not between good and poor sleepers. However, the findings of this study contradict this assumption. The rationale behind this phenomenon is elucidated through the utilized measuring instrument (SHI [37]), wherein a mere two items pertain to regularity.

Sleep disorders are known to be related to depression and anxiety, with bidirectional causality [57,58,59,60]. Importantly, shift workers also show higher levels of depression, anxiety, irritability, nervousness, suicidality, and somatization [10]. Some authors assume insomnia to be a mediator [5,16,57] for these complaints. Our data confirmed that, as expected, anxiety and depression are also relevant to the sleep of shift workers.

General predisposing personality traits of insomnia include perfectionism, general dissatisfaction, anxiety sensitivity, obsessive worrying, hypochondriacal concerns, internalization, and neuroticism [10,57,58,60,61].

In shift workers, the concept of shift work tolerance, i.e., the ability to adapt to shift work without negative consequences [62], is critical. Shift work intolerance has been shown to lead to insomnia and sleepiness, the use of sleeping pills, aggression, irritability, and gastrointestinal complaints [63]. Predisposing factors for shift work intolerance are considered to be female sex, older age, and early chronotype [64]. Resilience factors, on the other hand, include a lower degree of inertia and neuroticism, as well as a higher degree of flexibility, extraversion, and a more internal locus of control [62]. Those of these factors that we investigated yielded significant results, with two surprising exceptions, namely perfectionism and early chronotype. The underlying reasons for this remain unclear and require further investigation.

A Canadian survey [12] identified a clear association between shift work and burnout, as they reported that both shift and weekend workers exhibited significantly higher overall values for burnout and emotional exhaustion in comparison to a control group [44]. In addition to emotional exhaustion, irritability, depression and anxiety, burnout is also characterized by sleep disturbances, impaired recovery, and persistent fatigue [45]. The parallels with the consequences of shift work are noteworthy.

The effort-reward imbalance model [65,66] states that the perceived effort invested in the workplace should be in balance with the rewards received. The capacity of psychological detachment is pivotal in determining whether occupational or social stress at work has a negative impact on sleep [67], “affective rumination” can have a detrimental impact on sleep [51].

The findings substantiate the applicability of this model to the demographic of shift workers, albeit not to the extent that had initially been anticipated.

Attitudes toward shift work have also been identified as a significant factor influencing sleep. Individuals who express dissatisfaction with their work hours tend to exhibit diminished sleep quality, despite their objective sleep duration remaining unaltered [68]. Conversely, characteristics of the shift schedule (e.g., length of shifts, rotation), as well as the consequences of shift work (sleep disturbances, social difficulties), are predictors of attitudes toward shift work [69].

The unconventional work hours in shift workers have been shown to lead to “social desynchronization” within the private context, which hinders participation in social activities due to work shifts [13]. Consequently, a greater proportion of the shift worker population experiences elevated levels of social isolation [8]. Additionally, a higher percentage of shift workers are unmarried [57]. In shift workers, also more singles (53.2%) report sleep problems than those in a relationship (32.8%). Among day workers, no difference was found in terms of sleep problems regarding relational status [70].

Social stressors in the workplace are particularly detrimental [70], because unsatisfied needs for belonging and self-esteem protection can lead to a range of psychological impairments, including sleep problems [67].

Surprisingly, the findings reveal that there is no substantial discrepancy between day and shift workers with respect to the variables under consideration, with the exception of total sleep time, which exhibited a modest effect. The validity of these results was confirmed in subsequent multiple group comparisons. The pairwise post hoc tests (superscripted letters in Table 4) confirmed that the observed significance was attributable to the differences between good and poor sleepers, rather than between day and shift workers. However, there are compelling reasons for this: Due to the controlled participant recruitment and the median dichotomization based on the severity of insomnia, it is not possible to derive either prevalence or risk from the group size. Notably, the largest groups are day workers with adequate sleep (*n* = 65) and shift workers with poor sleep (*n* = 64, Table 1).

The distribution of the highest/lowest mean values across the four groups shows a clear pattern. For most characteristics, poor sleepers show tendencies toward greater psychological stress/poorer well-being, while good sleepers show the opposite (Table 4). It was not expected that shift workers would be more vulnerable, but rather that shift work would be associated with a higher risk [10,15,16]—while this risk cannot be assessed with this study design and was not the objective either.

This lack of difference, therefore, has no consequences for our project to develop a therapy for shift workers. The aim of the present study was to empirically verify whether the factors are also relevant for shift workers, not whether this is exclusively the case. Our main arguments that standard CBT-I is difficult to implement for shift workers and that interventions aimed at regularity could impair compliance remain valid.

However, the absence of these group differences may represent a kind of self-selection [71]: people who tolerate shift work less well may return to regular working hours. However, the same source also states that some people are forced into shift work for financial (shift allowances) or organizational reasons (e.g., childcare arrangements). In our sample, the primary reason given for “involuntary” shift work (27 people) was the necessity within the respective occupation.

Summarizing the results from questions 1 and 2, the hypothesis that the characteristics examined are indeed negatively associated with sleep (with the exception of perfectionism, chronotype, and the importance of sleep), and that this also applies to shift workers, can be considered confirmed.

The results of the regression analyses indicate that the independent variables, dysfunctional beliefs about sleep, cognitive arousal before bedtime, depression symptoms, and emotional instability in shift workers make the greatest contribution to explaining the variance. This finding does not contradict the results from questions 1 and 2; however, it does not correspond to the expectation that, for example, attitudes toward shift work, anxiety symptoms, or the characteristics of worry and tension would also play a greater role. The observed discrepancy between the groups in the regression models is noteworthy. In particular, it was observed that ERI imbalance is relevant exclusively for day workers. Since the results for the factors of the ERI model showed only small effect sizes across all analyses, this approach will not be pursued further.

What does all this mean for the overall project?

An overview of all the results of this study, including consideration of the respective effect sizes, leads to the following conclusion:

Perfectionism, chronotype, and the ERI model appear to have little or no significance for the sleep of shift workers. All other factors examined are relevant to the planned therapy and will therefore be integrated. The components of the new therapy are as follows:

Psychoeducation on the development and maintenance of sleep disorders, with a particular focus on the effects of excessive attention to sleep and the intention to fall asleep. Current tips for shift workers (e.g., [72] can also be presented. Attitudes toward shift work should also be discussed. Sleep hygiene and stimulus control can be presented, but without the rules on regularity. Other components should include classic interventions for affective disorders, e.g., establishing positive activities and a daily structure (e.g., planning time for friends, for oneself, sports, etc., regardless of regular times), which should also help to reduce feelings of social isolation. And, of course, depressive thought patterns should be examined and restructured if necessary. A larger part should also be devoted to interventions for anxiety disorders/worry, again using classic CBT-I methods such as reality check, decatastrophizing, and worry confrontation.

The therapy could be supplemented by methods from positive psychotherapy (happiness journal), mindfulness, and acceptance.

**Limitations.** The most important limitation is that this study was solely an online survey with a not stratified ad hoc sample, and all data are self-reported. The collection of objective data, including polysomnography and actigraphy recordings, was precluded due to the ongoing effects and after-effects of the SARS-CoV-2 pandemic at the time of data collection. The acquisition of participants via companies and physicians proved, nevertheless, challenging during this period. Due to the online format of the study, sleep problems as well as pre-existing conditions could only be assessed on the basis of information provided by the participants themselves. The survey instrument, which required a considerable 30 min to complete, may have introduced selection bias, as only those who were particularly attentive would have completed it.

The results of the regression analyses should not be overinterpreted, as they were calculated using numerous independent variables and the inclusion method (forced entry, forward) is not without controversy [73,74].

Since the finished therapy manual was tested in a randomized controlled trial [44,75], this study design seemed acceptable despite its limitations.

**Strengths.** Contrary to the approach of many other studies, our investigation examined a range of characteristics in a cohort comprising diverse occupational groups. This approach addresses the valid criticism that the results of studies on single occupational groups (e.g., nurses) cannot be generalized [15]. The comparison of the four groups of good/poor sleepers and day/shift workers can also be considered as a strength, as it yielded some revealing results. Another notable strength of the present study is the inclusion of an attitude towards shift work component, which was required for future research [68,69].

## 5. Conclusions

The objective of this study was to evaluate presumed sleep-related factors in shift workers. Factors whose relevance was empirically supported were intended to replace regularity-based interventions in a tailored treatment manual.

The findings of this study have informed the development of an innovative therapeutic approach that departs from conventional insomnia treatment, where sleep is typically the central focus. Instead, this broader framework integrates additional dimensions, including depression, anxiety, daily structure (even under shift-work conditions), social integration, attention to sleep, and depressive thought patterns. This facilitates the substitution of interventions that necessitate adherence to regularity, and a program explicitly designed for this target group should also enhance compliance and reduce attrition rates.

## Figures and Tables

**Table 1:** Sample description: Group sizes and distribution measures of the sleep variables.

	Shift Work (*n* = 112)	Day Work (*n* = 113)
Good sleepers (*n* = 113)	*48*	*65*
Poor sleepers (*n* = 112)	*64*	*48*
ISI	*M* = 10.58, *SD* = 5.97	*M* = 8.70, *SD* = 5.90
PSQI total	*M* = 7.57, *SD* = 3.85	*M* = 6.58, *SD* = 3.61
SOL	*M* = 33.12, *SD* = 26.95	*M* = 26.54, *SD* = 20.31
TST	*M* = 6.06, *SD* = 1.19	*M* = 6.77, *SD* = 1.29
ESS	*M* = 9.21, *SD* = 4.28	*M* = 8.71, *SD* = 4.08

ISI: Insomnia severity, measured by insomnia severity index [31]; PSQI total: Sleep quality [32]; SOL: sleep onset latency in minutes (PSQI, item 2 [32]; TST: total sleep time in hours (PSQI, item 4 [32]); ESS: Daytime sleepiness, measured by Epworth sleepiness scale [33].

**Table 2:** Correlations (*r*, *p*) of examined factors with sleep variables, shift workers only (*n* = 112, *df* = 110).

	ISI	PSQI Total	SOL	TST	ESS
HADS-D anxiety	0.53, 0.002	0.44, 0.002	0.34, 0.002	−0.15, 0.158	0.14, 0.174
HADS-D depression	0.58, 0.002	0.51, 0.002	0.51, 0.002	−0.21, 0.047	0.15, 0.158
HADS-D total	0.62, 0.002	0.53, 0.002	0.48, 0.002	−0.20, 0.053	0.15, 0.158
16 PF-R anxiety (global scale) ^1^	0.52, 0.002	0.50, 0.002	0.32, 0.002	−0.23, 0.022	0.30, 0.004
16 PF-R concern	0.45, 0.002	0.40, 0.002	0.28, 0.004	−0.14, 0.188	0.25, 0.013
16 PF-R emot. instability	0.58, 0.002	0.59, 0.002	0.44, 0.002	−0.33, 0.002	0.21, 0.040
16 PF-R tension	0.30, 0.002	0.30, 0.002	0.11, 0.284	−0.13, 0.205	0.25, 0.013
16 PF-R perfectionism	0.09, 0.387	0.00, 0.982	0.02, 0.845	−0.13, 0.223	0.12, 0.252
F-SozU social integration	−0.34, 0.002	−0.36, 0.002	−0.42, 0.002	0.08, 0.436	−0.04, 0.734
ERI Effort	0.21, 0.047	0.18, 0.080	0.15, 0.158	−0.06, 0.563	0.09, 0.408
ERI Reward	−0.35, 0.002	−0.29, 0.004	−0.16, 0.129	0.22, 0.033	−0.28, 0.006
ERI Overcommitment	0.37, 0.002	0.37, 0.002	0.26, 0.011	−0.19, 0.072	0.18, 0.089
ERI Imbalance	0.32, 0.002	0.27, 0.008	0.19, 0.072	−0.14, 0.171	0.20, 0.052
MZS	0.64, 0.002	0.50, 0.002	0.33, 0.002	−0.17, 0.095	0.30, 0.002
PSAS total	0.64, 0.002	0.64, 0.002	0.61, 0.002	−0.34, 0.002	0.20, 0.051
PSAS somatic	0.57, 0.002	0.53, 0.002	0.51, 0.002	−0.24, 0.019	0.13, 0.197
PSAS cognitive	0.56, 0.002	0.57, 0.002	0.56, 0.002	−0.30, 0.002	0.19, 0.064
rCSM	−0.08, 0.425	−0.20, 0.058	−0.30, 0.004	−0.00, 0.982	−0.06, 0.575
SHI ^1^	0.25, 0.014	0.30, 0.002	0.27, 0.008	−0.17, 0.093	0.22, 0.036
Importance of sleep	0.04, 0.368	0.05, 0.599	0.12, 0.254	0.19, 0.067	−0.01, 0.924
Like/dislike shiftwork ^2^	0.31, 0.002	0.23, 0.023	0.14, 0.170	−0.24, 0.019	0.14, 0.163

α-corrected [48,49]. ^1^ Pearson correlation, ^2^ biserial correlation. Background color: significant results. ISI: Insomnia severity, measured by insomnia severity index [31]; PSQI total: Sleep quality [32]; SOL: sleep onset latency in minutes (PSQI, item 2 [32]; TST: total sleep time in hours (PSQI, item 4 [32]); ESS: Daytime sleepiness, measured by Epworth sleepiness scale [33]; HADS-D: Hospital Anxiety and Depression Scale, German version [39], total score and subscales anxiety, depression; 16 PF-R: 16-Persönlichkeits-Faktoren-Test, revidierte Fassung [40], global scale anxiety, subscales concern, emotional instability, tension, perfectionism; F-SozU: Fragebogen zur Sozialen Unterstützung, subscale Social Integration [41]; ERI: Effort-Reward Imbalance Questionnaire [42], subscales effort, reward, overcommitment, imbalance; MZS: Meinungen-zum-Schlaf-Fragebogen (dysfunctional beliefs about sleep) [34]; PSAS: Pre-sleep arousal scale [36], total score, subscales somatic, cognitive; rCSM: reduced Composite Scale of Morningness [35]; SHI: sleep hygiene Index [37].

**Table 3:** Group differences (Mann–Whitney U Tests, good vs. poor sleepers).

Variable	Good Sleepers (*n* = 113)	Poor Sleepers (*n* = 112)	Test Statistic
	*M, SD*	*M, SD*	*(Z, p,* η^2^*)*
ISI	4.70, 2.51	14.62, 4.04	−12.95, 0.001, 0.75
PSQI	4.36, 1.83	9.80, 3.17	−11.46, 0.001, 0.58
SOL (min)	20.26, 14.79	39.46, 27.51	−6.13, 0.001, 0.17
TST (h)	7.11, 0.94	5.71, 1.21	−8.84, 0.001, 0.35
ESS	7.82, 3.42	10.10, 4.56	−3.82, 0.001, 0.07
HADS-D anxiety	5.24, 2.73	8.29, 4.03	−5.90, 0.001, 0.16
HADS-D depression	3.65, 3.14	6.68, 3.60	−6.50, 0.001, 0.19
HADS-D total	8.88, 4.97	14.96, 6.86	−6.98, 0.001, 0.22
16 PF-R anxiety (global scale)	61.12, 12.58	69.71, 13.63	−4.59, 0.001, 0.09
16 PF-R concern	22.73, 6.02	25.32, 6.18	−3.09, 0.002, 0.04
16 PF-R emot. instability	17.54, 4.16	21.04, 5.27	−4.96, 0.001, 0.11
16 PF-R tension	20.84, 5.21	23.35, 5.05	−3.57, 0.001, 0.06
16 PF-R perfectionism	23.00, 5.67	24.13, 5.02	−1.41, 0.170, 0.01
F-SozU social integration	47.29, 6.99	44.06, 7.52	−3.08, 0.003, 0.04
ERI Effort	7.85, 2.04	8.68, 2.27	−2.72, 0.008, 0.03
ERI Reward	20.94, 3.27	18.54, 4.02	−4.74, 0.001, 0.10
ERI Overcommitment	12.83, 3.25	14.62, 3.88	−3.67, 0.001, 0.06
ERI Imbalance	0.90, 0.30	1.16, 0.44	−4.72, 0.001, 0.10
MZS	54.35, 22.06	78.46, 26.46	−6.67, 0.001, 0.20
PSAS total	20.68, 5.57	30.84, 10.48	−7.77, 0.001, 0.27
PSAS somatic	9.65, 2.46	13.27, 4.67	−7.17, 0.001, 0.23
PSAS cognitive	11.03, 4.14	17.57, 7.28	−6.99, 0.001, 0.22
rCSM	19.25, 4.53	18.99, 4.03	−0.10, 0.923, <0.001
SHI	16.25, 6.56	18.53, 6.92	−2.33, 0.023, 0.02
like/dislike shiftwork (*n* = 112)	0.15, 0.36 (*n* = 48)	0.44, 0.50 (*n* = 64)	−3.28, 0.001, 0.10
Importance of sleep	3.20, 0.89	3.39, 0.74	−1.85, 0.065, 0.02

α-corrected [48,49]. Background color: significant results. ISI: Insomnia severity, measured by insomnia severity index [31]; PSQI total: Sleep quality [32]; SOL: sleep onset latency in minutes (PSQI, item 2 [32]; TST: total sleep time in hours (PSQI, item 4 [32]); ESS: Daytime sleepiness, measured by Epworth sleepiness scale [33]; HADS-D: Hospital Anxiety and Depression Scale, German version [39], total score and subscales anxiety, depression; 16 PF-R: 16-Persönlichkeits-Faktoren-Test, revidierte Fassung [40], global scale anxiety, subscales concern, emotional instability, tension, perfectionism; F-SozU: Fragebogen zur Sozialen Unterstützung, subscale Social Integration [41]; ERI: Effort-Reward Imbalance Questionnaire [42], subscales effort, reward, overcommitment, imbalance; MZS: Meinungen-zum-Schlaf-Fragebogen (dysfunctional beliefs about sleep) [34]; PSAS: Pre-sleep arousal scale [36], total score, subscales somatic, cognitive; rCSM: reduced Composite Scale of Morningness [35]; SHI: sleep hygiene Index [37].

**Table 4:** Multiple group comparisons between the four groups (Kruskal–Wallis *H*-Test, *df* = 3).

	*H, p,* η^2^	Groups: *M, SD*			
		Group 1: Poor Sleep Day Work *n* = 48	Group 2: Good Sleep Day Work *n* = 65	Group 3: Poor Sleep Shift Work *n* = 64	Group 4: Good Sleep Shift Work *n* = 48
ISI	168.00, 0.001, 0.75	14.33, 4.14 ^ab^	4.54, 2.63 ^b^	14.83, 3.99 ^a^	4.92, 2.35 ^b^
PSQI total	131.98, 0.001, 0.58	9.83, 2.96 ^ab^	4.17, 1.58 ^b^	9.78, 3.34 ^a^	4.62, 2.12 ^b^
SOL (min)	38.66, 0.001, 0.16	35.02, 23.87 ^a^	20.28, 14.48 ^b^	42.78, 29.71 ^ab^	20.23, 15.36 ^bc^
TST (h)	86.32, 0.001, 0.38	5.98, 1.34 ^ab^	7.35, 0.88 ^b^	5.51, 1.08 ^a^	6.79, 0.92 ^ab^
ESS	15.41, 0.003, 0.06	9.73, 4.44 ^ab^	7.95, 3.65 ^a^	10.37, 4.67 ^b^	7.65, 3.10 ^a^
HADS-D anxiety	40.28, 0.001, 0.17	9.35, 4.10 ^ab^	5.48, 2.72 ^b^	7.48, 3.82 ^a^	4.92, 2.74 ^b^
HADS-D depression	42.30, 0.001, 0.18	6.50, 3.51 ^ab^	3.68, 3.27 ^b^	6.81, 3.69 ^a^	3.60, 2.99 ^b^
HADS-D total	50.32, 0.001, 0.21	15.85, 6.67 ^ab^	9.15, 5.17 ^b^	14.30, 6.97 ^a^	8.52, 4.71 ^b^
16 PF-R anxiety (global scale)	27.68, 0.001, 0.11	71.90, 13.29 ^ab^	63.60, 11.23 ^a^	68.08, 13.76 ^a^	57.75, 13.60 ^b^
16 PF-R concern	20.72, 0.001, 0.08	26.54, 6.19 ^a^	24.18, 5.70 ^a^	24.41, 6.06 ^a^	20.77, 5.94 ^b^
16 PF-R emot. instability	30.82, 0.001, 0.13	21.25, 5.51 ^a^	18.43, 3.92 ^a^	20.89, 5.12 ^a^	16.33, 4.22 ^b^
16 PF-R tension	14.70, 0.003, 0.05	24.10, 4.57 ^a^	20.98, 4.47 ^b^	22.78, 5.34 ^b^	20.65, 6.12 ^b^
16 PF-R perfectionism	2.17, 0.559, −0.00	24.25, 4.77 ^a^	23.12, 6.13 ^a^	24.03, 5.24 ^a^	22.83, 5.03 ^a^
F-SozU social integration	17.98, 0.001, 0.07	42.42, 7.59 ^a^	46.12, 7.07 ^a^	45.30, 7.28 ^a^	48.87, 6.62 ^b^
ERI effort	10.01, 0.023, 0.03	8.31, 2.34 ^a^	7.63, 2.07 ^b^	8.95, 2.19 ^a^	8.15, 1.98 ^a^
ERI reward	23.22, 0.001, 0.09	18.87, 4.23 ^a^	20.82, 3.67 ^b^	18.30, 3.87 ^c^	21.10, 2.68 ^ab^
ERI overcommitment	15.52, 0.001, 0.06	14.88, 4.35 ^a^	13.20, 3.37 ^a^	14.42, 3.51 ^a^	12.33, 3.03 ^b^
ERI imbalance	23.72, 0.001, 0.09	1.10, 0.42 ^a^	0.89, 0.32 ^b^	1.21, 0.46 ^c^	0.92, 0.28 ^ac^
MZS	46.88, 0.001, 0.20	74.87, 28.00 ^ab^	56.43, 23.09 ^b^	81.14, 25.12 ^a^	51.54, 20.48 ^b^
PSAS total	62.87, 0.001, 0.27	32.21, 9.87 ^ab^	21.12, 5.51 ^b^	29.81, 10.88 ^a^	20.08, 5.65 ^b^
PSAS somatic	54.14, 0.001, 0.23	13.56, 4.67 ^ab^	9.80, 2.35 ^b^	13.05, 4.70 ^a^	9.46, 2.62 ^b^
PSAS cognitive	51.67, 0.001, 0.22	18.65, 6.91 ^ab^	11.32, 4.21 ^b^	16.77, 7.50 ^a^	10.63, 4.04 ^b^
rCSM	0.02, 0.999, −0.1	19.00, 4.50 ^a^	19.20, 4.78 ^a^	18.98, 3.68 ^a^	19.31, 4.22 ^a^
SHI	6.93, 0.084, 0.02	18.83, 6.03 ^a^	16.57, 7.22 ^a^	18.30, 7.57 ^a^	15.81, 5.60 ^a^
Importance of sleep	5.86, 0.129, 0.01	3.50, 0.72 ^a^	3.28, 0.82 ^a^	3.31, 0.75 ^a^	3.10, 0.97 ^a^

α-corrected [48,49]. Color coding: mean less/more pronounced in the direction of the risk factor. ^a, b, c, ab, ac, bc^: Groups sharing at least one letter are not significantly different; groups with no letters in common are significantly different. ISI: Insomnia severity, measured by insomnia severity index [31]; PSQI total: Sleep quality [32]; SOL: sleep onset latency in minutes (PSQI, item 2 [32]; TST: total sleep time in hours (PSQI, item 4 [32]); ESS: Daytime sleepiness, measured by Epworth sleepiness scale [33]; HADS-D: Hospital Anxiety and Depression Scale, German version [39], total score and subscales anxiety, depression; 16 PF-R: 16-Persönlichkeits-Faktoren-Test, revidierte Fassung [40], global scale anxiety, subscales concern, emotional instability, tension, perfectionism; F-SozU: Fragebogen zur Sozialen Unterstützung, subscale Social Integration [41]; ERI: Effort-Reward Imbalance Questionnaire [42], subscales effort, reward, overcommitment, imbalance; MZS: Meinungen-zum-Schlaf-Fragebogen (dysfunctional beliefs about sleep) [34]; PSAS: Pre-sleep arousal scale [36], total score, subscales somatic, cognitive; rCSM: reduced Composite Scale of Morningness [35]; SHI: sleep hygiene Index [37].

**Table 5:** Multiple linear regressions, forced entry, forward: Models and coefficients, separate for shift and day workers for the sleep variables.

ISI	Shift Workers						Day Workers					
Model	*R*^2^*_adj._* = 0.55, *F* (3, 108) = 46.64, *p* < 0.001						*R*^2^*_adj_*_._ = 0.46, *F* (3, 109) = 32.52, *p* < 0.001					
= −0.97 + 0.10 × (MZS) + 0.23 × (PSAS cognitive) + 0.30 × (HADS-D depression)							= −4.27 + 0.08 × (MZS) + 0.31 × (PSAS cognitive) + 3.35 × (ERI imbalance)					
coefficients	*b*	*SE*	*β*	*t*	*p*	*CI_95%_*	*b*	*SE*	*β*	*t*	*p*	*CI_95%_*
(constant)	−0.97	1.09		−0.89	0.377	−3.13; 1.20	−4.27	1.45		−2.94	0.004	−7.14; −1.39
MZS	0.10	0.02	0.45	5.95	<0.001	0.07; 0.13	0.08	0.02	0.37	4.72	<0.001	0.05; 0.12
PSAS cognitive	0.23	0.07	0.26	3.21	0.002	0.09; 0.37	0.31	0.07	0.35	4.44	<0.001	0.17; 0.45
HADS-D	0.30	0.13	0.19	2.23	0.028	0.03; 0.57						
ERI imbalance							3.35	1.12	0.21	2.99	0.003	1.13; 5.56
**PSQI total**	**Shift workers**						**Day workers**					
Model	*R*^2^*_adj._* = 0.47, *F* (3, 108) = *33.14*, *p* < 0.001						*R*^2^*_adj._* = 0.43, *F* (3, 109) = *28.55*, *p* < 0.001					
= −0.97 + 0.18 × (PSAS cognitive) + 0.19 × (emot. instability) + 0.04 × (dys. beliefs)							= −0.91+ 0.25 × (PSAS cognitive) + 0.03 × (MZS) + 2.03 × (ERI imbalance)					
coefficients	*b*	*SE*	*β*	*t*	*p*	*CI_95%_*	*b*	*SE*	*β*	*t*	*p*	*CI_95%_*
(constant)	−0.97	1.01		−0.97	0.336	−2.97; 1.02	−0.91	0.91		−1.00	0.322	−2.72; 0.90
PSAS cognitive	0.18	0.05	0.32	3.79	<0.001	0.09; 0.27	0.25	0.04	0.45	5.66	<0.001	0.16; 0.33
emot. instability	0.19	0.07	0.26	2.79	0.006	0.06; 0.33						
MZS	0.04	0.01	0.25	2.80	0.006	0.01; 0.06	0.03	0.01	0.22	2.76	0.007	0.01; 0.05
ERI imbalance							2.03	0.70	0.21	2.88	0.005	0.63; 3.42
**SOL (min)**	**Shift workers**						**Day workers**					
Model	*R*^2^*_adj._* = 0.39, *F* (2, 109) = *36.05*, *p* < 0.001						*R*^2^*_adj._* = 0.11, *F* (1, 111) = *14.91*, *p* < 0.001					
Equation	= −1.53 + 1.89 × (PSAS cognitive) + 1.47 × (HADS-D depression)						= + 11.23 + 1.06 × (PSAS cognitive)					
coefficients	*b*	*SE*	*β*	*t*	*p*	*CI_95%_*	*b*	*SE*	*β*	*t*	*p*	*CI_95%_*
(constant)	−1.53	4.57		−0.34	0.738	−10.59; 7.53	11.23	4.36		2.58	0.011	2.60; 19.86
PSAS cognitive	1.89	0.36	0.49	5.19	<0.001	1.17; 2.61	1.06	0.28	0.34	3.86	<0.001	0.52; 1.61
HADS-D depression	1.47	0.67	0.20	2.18	0.031	0.13; 2.80						
**TST (h)**	**Shift workers**						**Day workers**					
Model	*R*^2^*_adj._* = 0.11, *F* (1, 110) = *14.51*, *p* < 0.001						*R*^2^*_adj._* = 0.07, *F* (1, 111) = 14.84, *p* = 0.002					
Equation	= +7.53 − 0.08 × (emot. instability)						= + 7.71 − 0.97 × (ERI imbalance)					
coefficients	*b*	*SE*	*β*	*t*	*p*	*CI_95%_*	*b*	*SE*	*β*	*t*	*p*	*CI_95%_*
(constant)	7.53	0.40		18.80	<0.001	6.74; 8.33	7.71	0.33		23.69	<0.001	7.07; 8.36
emot. instability	−0.078	0.02	−0.34	−3.81	<0.001	−0.12; −0.04						
ERI imbalance							−0.97	0.31	−0.28	−3.11	0.002	−1.58; −0.35
**ESS**	**Shift workers**						**Day workers**					
Model	*R*^2^*_adj._* = 0.11, *F* (1, 110) = 15.32, *p* < 0.001						*R*^2^*_adj._* = 0.15, *F* (1, 111) = *20.73*, *p* < 0.001					
Equation	= +5.47 + 0.06 × (MZS)						= +4.82 + 0.6 × (MZS)					
coefficients	*b*	*SE*	*β*	*t*	*p*	*CI_95%_*	*b*	*SE*	*β*	*t*	*p*	*CI_95%_*
(constant)	5.47	1.03		5.33	<0.001	3.44; 7.51	4.82	0.92		5.22	<0.001	2.99; 6.65
MZS	0.06	0.01	0.35	3.91	<0.001	0.03; 0.08	0.06	0.01	0.40	4.55	<0.001	0.03; 0.00

ISI: Insomnia severity, measured by insomnia severity index [31]; PSQI total: Sleep quality [32]; SOL: sleep onset latency in minutes (PSQI, item 2 [32]; TST: total sleep time in hours (PSQI, item 4 [32]); ESS: Daytime sleepiness, measured by Epworth sleepiness scale [33]; HADS-D: Hospital Anxiety and Depression Scale, German version [39], total score and subscales anxiety, depression; 16 PF-R: 16-Persönlichkeits-Faktoren-Test, revidierte Fassung [40], global scale anxiety, subscales concern, emotional instability, tension, perfectionism; ERI: Effort-Reward Imbalance Questionnaire [42], subscales effort, reward, overcommitment, imbalance; MZS: Meinungen-zum-Schlaf-Fragebogen (dysfunctional beliefs about sleep) [34]; PSAS: Pre-sleep arousal scale [51], total score, subscales somatic, cognitive.

## Data Availability

The raw data supporting the conclusions of this article will be made available by the authors on request.

## Abbreviations

The following table presents the abbreviations utilized in the manuscript. In instances where variables or tests are concerned, the middle column indicates the meaning of high values.

*Abb.*	*meaning*	
REM	Rapid-eye movement	
NREM2	Non-Rapid-eye movement sleep phase 2	
SWD	Shift work disorder	
CBT-I	Cognitive behavioral therapy for insomnia	
*Abb.*	*variable*	*Meaning of high values*
TST	Total sleep time	longer
SOL	Sleep onset latency	longer
SSQ	Subjective sleep quality	poorer
DS/ESS	Daytime sleepiness/Epworth sleepiness scale	more severe
ISI	Insomnia severity index	more severe
PSQI	Pittsburgh sleep quality index	poorer sleep
MZS	sleep-related dysfunctional beliefs	more pronounced
PSAS	Pre-sleep arousal scale	Higher arousal
SHI	Sleep hygiene index	Poorer sleep hygiene
HADS-D	Anxiety, Depression, Total	Poorer psychological well-being
16 PF-R	16-Persönlichkeits-Faktoren-Test Anxiety global O: concern C: emotional instability (recoded) Q4: tension Q3: Perfectionism	personality traits, more pronounced: more anxiety more concerned more instability more tension more perfectionism
ERI	Effort-reward-imbalance (occupational) ERI_Effort ERI_reward ERI_OvercommitmentERI_Imbalance: >1: more effort than reward; =1:1:1 balanced; <1: less effort than reward	More perceived effort More perceived reward Tendency to exert more effort when perceived reward is insufficient More perceived imbalance
F-SozU	Fragebogen zur sozialen Unterstützung	Better social integration
rCSM	Reduced composite scale of morningness	early chronotype
	Importance of sleep	Higher individual importance of sleep
